# Effect of the Nd:YAG laser on the micro-tensile bond strength of composite resin to dentin with hypersensitivity using different universal adhesives

**DOI:** 10.4317/jced.61493

**Published:** 2024-06-01

**Authors:** Salim Hamidi, Mina Kahyaie-Aghdam, Mohammad-Esmaeel Ebrahimi-Chaharom, Mahdi Abed-Kahnamouei, Fatemeh Dabaghi-Tabriz

**Affiliations:** 1 Assistant professor, Department of Operative Dentistry, Faculty of Dentistry, Urmia University of Medical Sciences, Urmia, Iran; 2 Assistant professor, Department of Operative Dentistry, Faculty of Dentistry, Tabriz University of Medical Sciences, Tabriz, Iran; 3 Professor, Department of Operative Dentistry, Faculty of Dentistry, Tabriz University of Medical Sciences, Tabriz, Iran; 4 Associate professor, Department of Operative Dentistry, Faculty of Dentistry, Tabriz University of Medical Sciences, Tabriz, Iran; 5 Associate professor, Department of Restorative Dentistry, Faculty of dentistry, Tabriz University of Medical Sciences, Tabriz, Iran

## Abstract

**Background:**

This study aimed to evaluate the effect of the Nd: YAG laser on the tensile bond strength of composite resin to dentin with hypersensitivity using different universal bonding systems.

**Material and Methods:**

After cutting the crown of 252 molars, buccal cervical enamel was removed at a thickness of approximately 2 mm, and 252 smooth dentin surfaces were exposed. Then they were etched with 0.5 M ethylenediaminetetraacetic acid (pH = 7.4) to stimulate hypersensitivity. The specimens were then randomly divided into 12 groups (n= 21) according to the surface treatments performed. After etching and bonding according to the manufacturer’s recommendations in each group, the entire dentin surface was restored with Valux Plus composite resin. The samples were thermocycled and a universal test machine was used to measure the microtensile bond strength. The failure mode for each sample was observed under a stereomicroscope. For data analysis, the Bonferroni test, the independent t-test and the three-way anova test were used.

**Results:**

The average microtensile bond strength in non-laser samples was higher than the average in laser samples (*P*<0.001). In comparison with the bonding agent type in both cases with and without laser, the highest average microtensile bond strength was related to ALL-BOND (*P*<0.001), meanwhile the lowest average tensile strength in samples without laser was related to G-Premio universal adhesive (*P*<0.001), and the lowest average microtensile strength in samples with laser belonged to Prime and Bond Elect group (*P*<0.001).

**Conclusions:**

Nd: YAG laser irradiation of the dentin surface before applying the adhesive significantly decreased the microtensile bond strength of the composite resin to the dentin surface.

** Key words:**Nd:YAG laser, Bond strength, Dentin, hypersensitivity, Universal adhesives.

## Introduction

A longer lifespan of teeth in the oral cavity increases the odds of noncarious cervical lesions, including erosion, abrasion, and abfraction. As a result, dentin hypersensitivity (DH) is a common complaint among adults and is considered one of the most critical and painful conditions in dentistry (1). DH occurs when dentinal tubules are exposed to the oral cavity in teeth with gingival recession (2,3).

Various techniques are available for the treatment of DH, including instructions for proper brushing, dietary advice, occlusal adjustment, application of desensitizing products/salts (potassium ions, oxalate, sodium fluoride), irradiation of low-power or high-power lasers, the use of adhesive systems, and adhesive restorations (3,4).

Laser therapy has been established as an effective method to reduce DH. Among different laser types, the mode of action of the Nd: YAG laser has been highlighted. This laser has been used since 1985 and has shown the potential to obliterate the dentinal tubules through a process called ‘melting and resolidification without causing pulpal damage or cracks when used with an appropriate protocol. In addition, The Nd:YAG laser can produce a sealing depth of 4 mm in dentinal tubules, which usually causes an immediate reduction in dentin hypersensitivity. Brief exposure to Nd:YAG laser beams may be applied to fuse dentin, and the dentin fused in this manner becomes solid with a glazed, nonporous surface (5).

The effect of desensitizers on the bond strength of adhesive restorations is controversial. Dentin surfaces were less favorable bonding substrates after using desensitizing agents (6). In recent years, researchers have used lasers to promote adhesive bond strengths (7). Several studies have shown that dentin irradiation with Nd:YAG laser beams before adhesive procedures decreases the bond strength of resin composites. This effect is brought about by the obliteration of the dentinal tubules due to the melting and resolidification of the irradiated dentin (8-10). However, Nd:YAG laser irradiation over the adhesive and prior to polymerization leads to positive results due to the warming of dentin (below its melting point); therefore, better penetration of the adhesive might occur (11-13).

The effects of laser irradiation on dentin with different universal adhesives have not yet been adequately evaluated.

However, this study aimed to evaluate the effect of Nd: YAG laser on the tensile bond strength of composite resin to dentin using different universal bonding systems.

## Material and Methods

**Figure 1:**
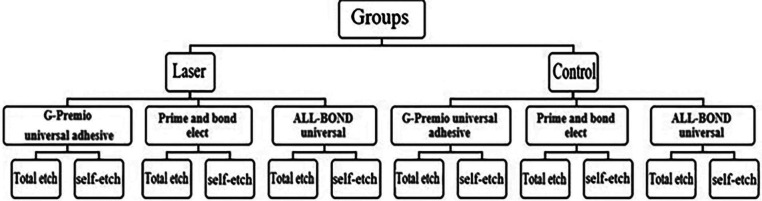
Division of groups.

252 molars were used in this study. They were examined using a loup, and only teeth without caries, fractures, or cracks were selected for use. After removing debris from teeth, they were stored in sterile saline (0.9% NaCl) until use. After cutting the crown of the teeth, buccal cervical enamel was removed with a water-cooling diamond disc (Isomet 11-1180; Buehler Ltd, Evanston, IL) at a thickness of approximately 2 mm, and 252 smooth dentin surfaces were exposed. Then they were mounted in an acrylic mold. The dentin surfaces of the samples were etched with 0.5 M ethylenediaminetetraacetic acid (EDTA) (pH = 7.4) for 2 min to stimulate hypersensitivity and then washed with distilled water for 30 s and dried. The samples were then rinsed with an air-water syringe for 30 s and dried. The specimens were then randomly divided into 12 groups (n= 21) according to the surface treatments performed (14), (Fig. 1).

The samples were randomly divided into two substrate groups based on the use or non-use of the laser. In the laser groups, the teeth were irradiated with Nd:YAG laser (Nd:YAG Dental Laser, Lambda Scientifica Srl, Vicenza, Italy) for a pulsed wavelength of 1.064 µm; non-contact with a distance of 1 mm from the surface; an output power of 1.5 W; an energy level of 50 mJ and a frequency of 15 Hz for 10 seconds. The fiber optic diameter was 400 µm (15).

After etching and bonding according to the manufacturer’s recommendations in each group ( Table 1), the entire dentin surface was restored with Valux Plus composite resin (3M ESPE, St Paul, MN, USA). The composite resin was applied on the entire surface of exposed dentin in 2 mm increment and light-cured (L.E. Demetron 1, SDS/Kerr, Orange, CA, USA) for 20 seconds at 1000 mc/cm2 intensity (16).

The samples were then immersed in 37°C water for 24 h. The samples were thermocycled 500 rounds at 5-55°C at a 10-second interval (14). Then each specimen was sectioned in a buccolingual and mesiodistal direction to prepare rectangular bars of resin-dentin with a mean area of 1 mm using a diamond disk (Isomet, Buehler Ltd, Lake Bluff, IL, USA) at low speed under water cooling. Bond strength in each group was measured using a microtensile tester (Bisco, Schaumburg, IL, USA) at a crosshead speed of 1 mm/min (16).

After performing the microtensile bond strength test, the failure patterns of the specimens were determined under a stereomicroscope (Nikon SMZ800, Tokoyo, Japan) at ×40 magnification.

Failure mode was classified into three types:

Type I: Cohesive failure in dentin

Type II: Cohesive failure in composite block

Type III: Adhesive failure

-Statistical procedures

In order to investigate the effect of using a laser, the type of bonding and the method of application of bonding on the strength of the tensile band, the normality of the data was checked at first. This work was done using the Kolmokov Smirnov test and the results showed that the tensile bond strength values have a normal distribution (*P*=0.200). Therefore, a three-way analysis of variance was used to compare the strength of the tensile band in the groups.

## Results

Comparison of microtensile bond strengths between groups by three-way ANOVA showed that the effects of laser, type of bonding agent, and bonding application were significant (*P*<0.001). Using these three variables, 84% of the changes in microtensile bond strength are predicTable. Furthermore, the cumulative effect of laser (*P*<0.001) and bonding application type (self-etch or total-etch) (*P*=0.025) was significant.

The Bonferroni test for comparison between two types of bonding agents also showed that their effects were significantly different (*P*<0.001) ( Table 2).

In general, the average microtensile bond strength in non-laser samples was higher than the average in laser samples. In comparison with the bonding agent type in both cases with and without laser, the highest average microtensile bond strength was related to ALL-BOND, but the lowest average tensile strength in samples without a laser was related to G-Premio universal adhesive, and the lowest average microtensile strength in samples with laser belonged to the Prime and Bond Elect group.

**Figure 2:**
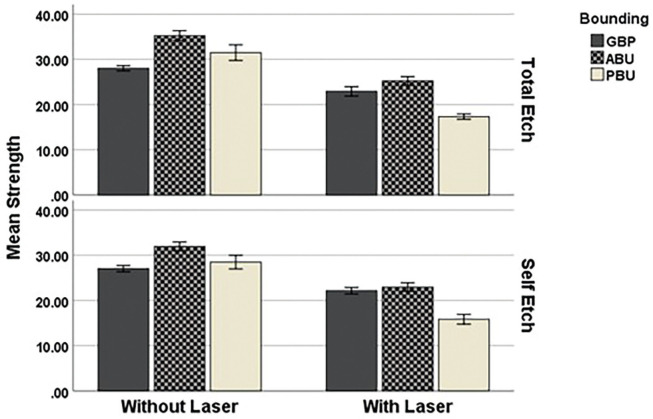
Mean tensile bond strength in all group.

As shown in  Table 3 and Figure 2, the highest mean microtensile bond strength in the non-laser group was related to ALL-BOND with an average of 33.59±2.86 MPa, and the lowest average was related to G-Premio universal adhesive with an average of 27.51±1.51 MPa. Also, in the non-laser group, among the mode of bonding application (self-etch or total-etch), the highest average was related to ALL-BOND bonding agent in the total-etch mode (35.22±2.51 MPa), and the lowest average was related to G-Premio universal adhesive in the self-etch mode with an average of 27.2±1.57 MPa.

In the group with laser, the highest mean microtensile strength in all types of bonding was related to ALL-BOND with an average of 24.09±2.41 MPa, and the lowest average was related to Prime and Bond Elect with an average of 16.57±2.11 MPa. In addition, among the bonding application modes, the highest average was related to ALL-BOND bonding in the total-etch mode with an average of 25.22±2.15 MPa, and the lowest average was related to Prime and Bond Elect bonding agent in the self-etch mode with an average of 15.84±2.46 MPa.

Also, an independent t-test was used to compare the average microtensile bond strength by bonding agent type and etching mode between samples with and without a laser. The results showed a statistically significant difference between the mean microtensile bond strength of all the samples with and without laser in both groups (*P*<0.001).

This comparison was also performed separately in each type of bonding, which was also significant (*P*<0.001).

Comparing the microtensile bond strength between self-etch and total-etch samples between the two groups showed only the samples belonging to the laser group with G-Premio universal adhesive had a non-significant difference between self-etch and total-etch modes (*P*=0.217).

**Figure 3:**
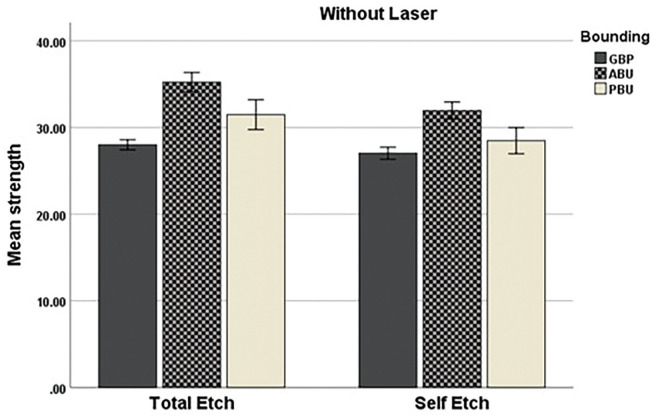
Comparison of tensile bond strengths in laser samples.

**Figure 4:**
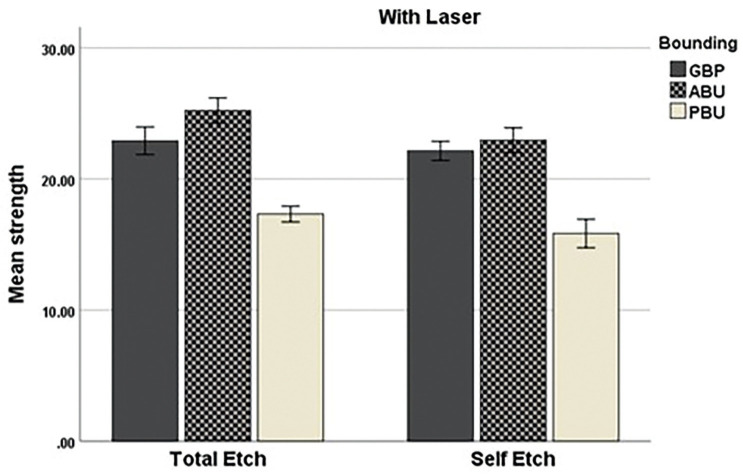
Comparison of microtensile bond strengths in non-laser samples.Comparison of microtensile bond strengths in non-laser samples.

Figures 3 and 4 show a comparison of microtensile bond strengths between the groups.

The failure modes of the samples are presented in  Table 4, which shows that all failures in the laser group were of the adhesive type. In the non-laser group, only in the two groups of G-Premio SE and TE, all the failure modes were of the adhesive type. In the ALL-BOND TE group, 85% of failure modes were reported as adhesive, 5% as cohesive, and 10% as mixed.

In the ALL-BOND SE group, 90% of failures were reported as adhesive, 5% as cohesive, and 5% as mixed. Also, in the Prime-&-Bond TE group, 90% of failures were adhesive, and 10% were mixed. In the Prime-&-Bond SE group, 95% of the failures were of the adhesive type and only 5% were of the mixed type.

## Discussion

In comparison with enamel, bonding to dentin presents a much greater challenge. Adhesive materials can interact with dentin in different ways—mechanically, chemically, or both. Dentin adhesion relies primarily on the penetration of adhesive monomers into the network of collagen fibrils exposed by acid etching. Today, different methods are available for dentin etching (conditioning), such as different etchant solutions, air abrasion, or lasers (Er:YAG and Nd:YAG) (10,19).

The mechanism of the Nd:YAG laser’s effect on dentin relies on thermal energy absorption (19). The hydroxyapatite crystals of dentin melt partly or completely, move, and increase in size once the activation energy is sufficient. Finally, the dentinal tubules become occluded, with a sealing depth of 4 µm (5).

The Nd: YAG laser treats hypersensitivity by narrowing the dentin tubules, which may inhibit adhesion between resin and dentin. SEM observations (thin resin tags and separations in the hybrid layer) supported the lower bond strength values in the laser group (20,21).

With the advent of universal adhesives, dentists are now using the same dentin adhesive for different adhesive strategies (i.e., self-etch, etch-and-rinse, or selective enamel etch adhesive), according to each specific clinical situation. Because of their multi-strategy approach, this new generation of one-bottle dental adhesives has become very popular in dentistry (22).

The use of Nd:YAG laser before the adhesive restorative process is not recommended since this type of irradiation leads to tissue depletion in dentinal tubules with their partial or total occlusion, making adhesive infiltration and micromechanical retention more difficult (23,24).

However, Nd:YAG laser after adhesive application, as has already been proposed by some researchers, seems a more appealing way to achieve higher bond strengths (12,23).

According to the results reported by Frankle *et al*. , low energy densities (on the order of 5 J/cm2) induce heating of the dentin (below its melting point), allowing for better adhesive penetration, which, in turn, results in higher bond strengths. On the other hand, if the energy applied is too high (50 J/cm2), bond strength is reduced (13).

Gan *et al*. studied the effect of Nd:YAG laser irradiation on long-term bond strength with etch-and-rinse adhesives and concluded that laser irradiation increased dentin bond durability (25).

Liu *et al*. investigated the microtensile bond strength of the self-etch adhesive to dentin and concluded that pulsed Nd:YAG laser irradiation could increase the bond strength (26).

In our study, the effect of Nd: YAG laser irradiation on the microtensile bond strength of universal adhesives on the dentin surface was evaluated. In general, the average microtensile bond strength in non-laser samples was higher than in the laser samples.

The findings of this study about the decrease in microtensile bond strength in laser samples are in contrast with Jafarnia *et al*. study, in which it was suggested that laser application results in irregularities in dentin or enamel, leading to increased bond strength (27).

The findings of this study about the decrease in microtensile bond strength in laser samples are consistent with that of Arisu *et al*. , who concluded that occluding dentinal tubules by laser application can decrease the bond strength (28).

Jafarnia *et al*. studied the effect of Nd:YAG laser irradiation on microtensile bond strength of universal adhesives to dentin and concluded that Nd:YAG laser irradiation on the dentin surface before adhesive application with G-Premio and Clearfil S3 Bond adhesives significantly increased the microtensile bond strength of the restoration to the dentin surface (27).

Differences in the results of studies can be attributed to several factors, such as the methodology, type of adhesive systems used, laser parameters, and the type of the bond strength test.

In current study comparing the types of bonding showed that the highest average microtensile bond strength in non-laser samples was related to ALL-BOND, with the lowest average tensile strength in the G-Premio universal adhesive. In the laser samples, the highest average tensile strength was related to ALL-BOND, with the lowest average tensile strength in the Prime-and-Bond Elect adhesive. G-Premio bond strength in the non-laser group was the lowest in comparison with other bonding agents, but in the laser group, the lowest bond strength was related to the Prime-and-Bond Elect adhesive, indicating that G-Premio adhesive exhibited better results in the laser group in comparison with the non-laser group. Therefore, in conclusion, because of the higher acidity of G-Premio adhesive (pH=1.5) in comparison with ALL-BOND (pH=3.1-3.2) and Prime-and-Bond Elect adhesive (pH=2.5), And considering the role that the acidity of an adhesive can have in partial or complete removal of the smear plug blocking the dentin tubule and exposing the thin and dense strands of collagen fibrils for the purpose of increasing the strength of the laboratory bond (22), it can be concluded that G-Premio due to The stronger acidity itself opens the blocked tubules in the laser samples more effectively and makes the dentine surface more effectively receptive to the adhesive layer. G-Premio bond could open the occluded tubules in laser samples more effectively.

According to the results, Nd: YAG laser irradiation significantly affected the microtensile bond strength of the adhesive to the dentin surface.

In addition to the acidity, application technique, adhesive composition, monomers and fillers, and the percentage of each component play an important role in bonding quality. Therefore, differences in the results between adhesives in laser or non-laser groups could be interpreted based on their different properties.

The present *in vitro* study evaluated extracted teeth without simulating dentinal fluid pressure; therefore, it is difficult to compare the results with clinical conditions. In clinical conditions, when dentin is exposed to the oral cavity, dentinal fluid flows from the pulp to exposed dentin because of the interstitial fluid pressure in the pulp. Some studies have reported that dentinal fluid flow affects the diffusion of adhesive resins into dentinal tubules (25).

In addition, cervical cavities in the present study were surrounded by enamel that can affect composite resin polymerization shrinkage in restorative procedures. Therefore, in a sense, it had the benefit of eliminating the composite resin polymerization shrinkage effect. However, if we want to simulate clinical situations in future studies, we should prepare cervical cavities that are not surrounded by enamel walls.

## Conclusions

Nd: YAG laser irradiation of the dentin surface before applying the adhesive significantly decreased the microtensile bond strength of the restoration to the dentin surface. Comparison of the bonding agent type in both cases with and without laser showed the highest average microtensile bond strength with ALL-BOND adhesive, with the lowest average microtensile strength in samples without laser in the G-Premio universal adhesive. However, in the laser samples, the highest average microtensile bond strength was reported in the Prime and Bond Elect group.

## Figures and Tables

**Table 1:** Materials used in this study and application techniques of adhesive.

Material	PH	Type	Manufacturer	Composition	Application technique
G-Premio universal adhesive (GPB)	1.5	Total etch or self-etch	GC America	10-MDP, 4-META, MTDP, methacrylic acid ester, silica, acetone, water, photoinitiators	etch-and-rinse: acid etching (15s), rinsing (15s) and gently dry, two coats of adhesive applied (left for10 s after application) and air-drying (5s) under maximum air pressure, light-cured for 10s.^17^ self-etch: two coats of adhesive applied (left 10 s after application) and air-drying (5 s) under maximum air pressure, light-cured for 10 s.^17^
Prime and bond elect (PBE)	2.5	Total etch or self-etch	Dentsply Sirona, USA	PENTA P, methacrylate resins, HEMA, Acetone, Water, Initiators	etch-and-rinse: acid etching (15 s), rinsing (15 s), blot-drying, two coats of adhesive applied (20 s) and moderate air stream drying (5 s), light-cured for 10s.^17^ Self-Etch: two-coats of adhesive applied (20 s) and moderate air stream drying (5 s), light-cured for 10 s.^17^
ALL-BOND universal (ABU)	3.1	Total etch or self-etch	Bisco, Schaumburg IL, USA	10-MDP, 2-HEMA, BisGMA, ethanol, water, photoinitiator	etch-and-rinse: acid etching (15 s), rinsing (15 s), blot-drying, two coats of adhesive applied (10-15 s) and thorough air-drying (10 s), light-cured for 10 s.^18^ self-etch: two coats of adhesive applied (10-15 s) and thoroughly air-drying (10 s), light-cured for 10 s.^18^
Valux Plus composite			3M ESPE, St Paul, MN, USA	Bis-GMA and TEGDMA,zirconia/silica fillers 66% by volume with a particle size of 0.01-3.5 µm	

**Table 2:** Comparison of bonding types - Bonferroni test.

Bounding agent		Mean Difference	P-value
ALL-BOND	Prime and Bond Elect	5.56	<0.001
	G-Premio universal adhesive	3.82	<0.001
Prime and bond elect	G-Premio universal adhesive	-1.73	<0.001
	Self-etch	22.95±2.13	
	Total-etch	25.22±2.15	

**Table 3:** Mean microtensile bond strengths in all the groups.

Laser	Bonding	Etching
No	G-Premio universal adhesive	Total	27.51±1.51
		Self-etch	27.2±1.57
		Total-etch	28±1.31
	ALL-BOND	Total	33.59±2.86
		Self-etch	31.95±2.21
		Total-etch	35.22±2.51
	Prime and Bond Elect	Total	29.97±3.91
		Self-etch	28.47±3.41
		Total-etch	31.47±3.88
Yes	G-Premio universal adhesive	Total	22.52±2.37
		Self-etch	22.13±1.64
		Total-etch	22.91±2.37
	ALL-BOND	Total	24.09±2.41
		Self-etch	22.95±2.13
		Total-etch	25.22±2.15
	Prime and bond elect	Total	16.57±2.11
		Self-etch	15.84±2.46
		Total-etch	17.31±1.35

**Table 4:** Numbers and percentages of failure modes after adhesive application in each group.

	Non-laser						Laser					
	G-PREMIO TE	G-PREMIO SE	ALL-BOND TE	ALL-BOND SE	PRIME-&-BOND TE	PRIME-&-BOND SE	G-PREMIO TE	G-PREMIO SE	ALL-BOND TE	ALL-BOND SE	PRIME-&-BOND SE	PRIME-&-BOND SE
Adhesive	100%	100% (21)	85% (18)	90% (19)	90% (19)	95% (20)	100%	100%	100%	100%	100%	100%
Cohesive	0%	0%	5% (1)	5% (1)	0%	0%	0%	0%	0%	0%	0%	0%
mixed	0%	0%	10% (2)	5% (1)	10% (2)	5% (1)	0%	0%	0%	0%	0%	0%
Total	21	21	21	21	21	21	21	21	21	21	21	21

## Data Availability

The datasets used and/or analyzed during the current study are available from the corresponding author.
